# Triple enzymatic immunochemistry for interneuron populations in postmortem human cerebral cortex

**DOI:** 10.1016/j.heliyon.2023.e20626

**Published:** 2023-10-11

**Authors:** Pablo Juarez, Verónica Martínez-Cerdeño

**Affiliations:** a Institute for Pediatric Regenerative Medicine (IPRM), Shriner's Hospital for Children and UC Davis School of Medicine, Sacramento, CA, USA; b Department of Pathology & Laboratory Medicine, UC Davis School of Medicine, Sacramento, CA, USA; c MIND Institute, UC Davis School of Medicine, Sacramento, CA, USA

## Abstract

Immunostaining is an antibody-based tool used to visualize proteins in tissue. Enzymes or fluorochromes conjugated to antibodies are used to detect proteins of interests. Fluorescent immunostaining can be used in human tissue, however due to the high autofluorescence of non-perfused human tissue, enzymatic immunostaining is better suited. Enzymes produce a colored product that is detectable by light microscopes. Here we describe a successful triple immunochemistry protocol to enzymatically label three distinct populations of interneurons (Parvalbumin+, Calbindin+, and Calretinin + interneurons) in non-perfused formalin fixed human brain cerebral cortex. Signal was achieved using a combination of horseradish peroxidase (HRP) and Alkaline Phosphatase (AP) enzymes and color was generated using the insoluble chromogens: 3,3′- Diaminobenzidine (DAB, Brown), Vector Blue (Blue), and Vector VIP (Pink). There were no noticeable background and minimal signal overlap between the different colors. We were able to successfully stain human cortical tissue and distinguish morphological properties of the three interneuron (IN) populations.

## 1 Introduction

Immunohistochemistry (IHC) is an essential and reliable technique commonly used in many laboratories for research and clinical diagnosis purposes. There are multiple forms of IHC that allow antigen detection on human tissue, including Immunofluorescence (IF) and Enzymatic based techniques. Human tissue used for IHC is not perfused and routinely fixed in 10 % neutral-buffered formalin, leading to appreciable levels of autofluorescence when using an IF based technique. This among other things, such as photobleaching, limits the use of IF in formalin fixed human tissue, making enzymatic based IHC a more suitable approach for overcoming these challenges. Here we describe a successful triple IHC method that we developed for staining three antigens in non-perfused formalin fixed human brain tissue. We present a detailed overview of the different steps, advantages and limitations involved with using our triple enzymatic based IHC method to achieve successful quantification of three different cell populations in the human brain.

IHC uses antibodies to detect specific proteins in fixed tissue. An antibody, also known as an immunoglobulin, is a blood protein produced to recognize and bind to a specific portion of a protein called an antigen. There are 5 antibody isotypes that are classified according to their heavy chain (Ig): IgG, IgM, IgA, IgD and IgE. Of these isotypes, IgG is the most abundant in normal human serum, accounting for 70–85 % of the total antibody pool [1]. The IgG isotype is also the most used in research due to its high abundance and high specificity towards antigens. Monoclonal and Polyclonal antibodies are two types of antibodies that contain a mixture of immunoglobulin molecules against single or multiple epitopes of an antigen. Polyclonal antibodies are specific against multiple epitopes in a specific antigen and are produced by administrating a laboratory animal like a mouse, rabbit, or goat, with a specific antigen. Different B cell clones in an animal produce a heterogenous pool of polyclonal antibodies characterized by having small alterations in the F_ab_ binding region of each antibody, the paratope [2]. Small alterations in the paratope allow for high affinity recognition and binding to different epitopes on a target antigen that differ by a small number of amino acids. Manufacturing of a homogenous population of monoclonal antibodies is a more sophisticated process that involves *ex-vivo* fusing of spleen cells with myeloma cells to make antibody producing hybridomas. Functionally, monoclonal antibodies have monovalent affinity that allows them to recognize a single epitope on an antigen.

IHC can be used directly or indirectly to detect target proteins. For direct detection, the F_C_ region of the primary antibody is conjugated to an enzyme or fluorescent molecule that produce color. In fluorescent IHC, a fluorescent dye or fluorochrome is used to visualize the site of antibody binding. Fluorochromes are photosensitive chemical compounds that absorb light energy of a specific wavelength and emit light at a longer wavelength. In the enzymatic IHC, a chemical reaction is catalyzed by an enzyme to form a colored insoluble precipitate at the site of antibody binding. Horseradish Peroxidase (HRP) and Alkaline Phosphatase (AP) are detection enzymes commonly used. The HRP catalyzes a soluble substrate like 3,3′-Diaminobenzidine (DAB), in the presence of an activator (H_2_O_2_), into a brown insoluble product. Other HRP substrates can also be used, such as Vector NovaRED or Vector VIP that produce red or purple precipitate products, respectively. Another commonly used enzyme is AP that can catalyze a reaction on multiple substrates to produce different colors. For example, Vector Blue transforms into a blue insoluble precipitate in the presence of the AP enzyme.

Signal can be further increased using amplification methods such as Avidin-Biotin Complex (ABC), Labeled Streptavidin-Biotin (LSAB), and Polymer-Based amplification. With the ABC detection method, the secondary antibody is conjugated to a biotin molecule, that in turn binds to avidin molecules that are linked to a color producing enzyme molecule, most commonly HRP [[3], [4], [5]]. This results in a high enzyme:antibody ratio and a strong signal amplification. The system described above can also be used with fluorescent molecules where a biotin conjugated secondary antibody links the primary antibody to fluorochrome-conjugated avidin molecules. Non-biotin amplification methods, like VectaFluor Excel Amplified Fluorescent Staining System (Vector Laboratories, Burlingname, CA, USA), where the primary antibody binds to multiple dye-conjugated antibodies, can also be used to strengthen fluorescence signal.

IHC can be used for detecting a single antigen or multiple antigens in the same tissue. Multiple IHC involves the use of different antibodies conjugated to different fluorochromes or enzymes. For multiple fluorescent IHC, two or more antibodies conjugated to different fluorochromes are required to produce distinct color signals. The large number of commercially available fluorochromes allow for simultaneous detection of multiple antigens. In a fluorescence microscope, a specimen is excited with a specific wavelength of light and emits a longer wavelength that allows us to visualize an antigen of interest. The number of colors that can be detected simultaneously relies on the use of fluorochromes with non-overlapping excitation and emission wavelengths. For example, the emission wavelength for FITC (520 nm, green) and Rhodamine (627 nm, red) are practically non-overlapping allowing for accurate color differentiation for multiple antigens tagged with these fluorochromes.

For enzymatic IHC, double labeling can also be achieved by using antibodies that are developed using two different enzymes, for example HRP and AP. Each of these enzymes catalyze independent reactions using different substrates to produce different color precipitates. There are several commercially available enzyme substrates that yield unique colors when developed with an HRP or AP based reaction. Successful enzymatic procedure relies on choosing enzymatic substrates that will produce distinct and contrasting colors. For example, combining a HRP based-DAB reaction with an AP-based Vector blue reaction to yield brown and blue color precipitates, respectively. Detecting a third antigen using enzymatic based IHC is possible but difficult and requires using a specific protocol. Previous studies have successfully employed the use of a triple IHC technique on human oral mucosa, lymph node, and testicular tissue [6]. Moreover, studies commonly assess for changes between two distinct populations of cells within the human brain via IHC. However, to our knowledge, there is no other laboratory that actively uses a triple enzymatic staining protocol in human fixed brain samples to assess for changes in three cortical cell populations. Therefore, our aim was to develop a triple stain IHC protocol to successfully stain and quantify three distinct cortical inhibitory GABAergic interneuron populations (Parvalbumin (PV)+, Calbindin (CB)+ and Calretenin (CR)+) in formalin fixed human brain tissue. These three markers belong to the EF-hand family of the calcium binding proteins [7] and play a physiological role in intracellular calcium homeostasis, most notably by acting as Ca^2+^ transporters across the cell membrane or by acting as Ca^2+^ modulate sensors to regulate the amount of free Ca^2+^ in the cytosol of the cell [8]. In addition to having diverse kinetic properties, these three markers are useful for distinguishing three almost non-overlapping populations of cortical GABAergic interneurons. The detailed triple enzymatic staining protocol we describe here is the first successful protocol for staining and quantifying three distinct populations of cortical inhibitory GABAergic interneurons (Parvalbumin+, Calbindin+ and Calretinin+) in non-perfused formalin fixed human brain tissue.

## 2 Material and methods

Here we describe the triple enzymatic staining protocol to label three distinct populations of interneurons (Parvalbumin+, Calbindin+ and Calretinin + cells) in non-perfused formalin fixed human brain cerebral cortex.

### 2.1 Tissue Preparation and quantification

We collected human prefrontal cortex samples from Brodmann areas (BA's) 9, 46 and 47. The tissue was obtained from the Hispanoamerican Brain Bank of Neurodevelopmental Disorders [9]. The tissue was fixed in formalin for several months and then cryoprotected in 30 % sucrose with PBS, before embedding the tissue samples in optimal cutting tissue compound (OCT). We cut tissue blocks in a temperature-controlled cryostat (−20 °C) to obtain 14 μm-thick sections. The protocol was optimized for using multiple antibodies to stain slide mounted tissue. Using thin-cut slices to obtain a single layer of cells for quantification is ideal as it reduces quantification errors, such as inadvertently counting overlapping cell as only one cell. We used a 100× oil objective on a microscope (Olympus B×61 microscope with a Hamamatsu Camera, a Dell Precision PWS 690, Intel Xeon CPU Computer with Microsoft Windows XP Professional V.2002 system, and MBF Bioscience StereoInvestigator V.9 Software, MicroBrightField, Williston, VT) to quantify the number of immunopositive cells for each cortical inhibitory GABAergic interneuron subtype (PV+,CB+ and CR+) as has been previously described [10]. Briefly, within the selected regions of interest, we counted the total number of PV+,CB+ and CR+ cells within a 3-mm bin parallel to the pial surface and that spanned through the thickness of the cortical gray matter. Cell quantification was based on the number of immunopositive cells for each interneuron subtype (PV+,CB+ and CR+ cells) , and we counted cells as positive if those had immunostaining within the cytoplasm.

## 3 Method

### 3.1 Day 1: first primary antibody incubation

Tissue Preparation: To begin the triple straining procedure, ensure that tissue is at room temperature (RT). If the tissue is preserved in the freezer, thaw it at RT (20–22 °C) for 10 min. After thawing, carefully place up to 24 slides on a slide staining rack (2935M6 Medicus Health) equally spaced apart. Next perform the defatting process, which is essential for helping to permeabilize the tissue by removing surface lipids that may interfere with specific antibody binding. Immerse slides in a 1:1 solution of 100 % Ethanol and Chloroform at RT for 10 min. Follow this step by rehydrating the tissue in successive baths of 100 % ethanol (5 min), 90 % ethanol (5 min), 70 % ethanol (4 min), 50 % ethanol (3 min), Deionized Water (DiH_2_O, 30 s) and TBS (2x for 5 min each). Formalin-fixed tissue is characterized by the formation of methylene bridges that often mask antigenic sites and interfere with antibody binding [11]. Antigen retrieval is essential for breaking methylene bridges and helping expose antigen binding sites to obtain optimal signal. Note that this is a time and temperature sensitive step. Immerse slides in a 10 % DIVA Decloaker Antigen Retrieval buffer (DIVA) for 5 min. DIVA is a heat retrieval buffered solution used in heat-induced antigen retrieval of formalin-fixed tissues for IHC. Following this 5-min wash, place the slides in the decloaking chamber for 6 min at 100 °C (V.3.7.2.2). The Decloaking chamber is a specialized bench top temperature pressure chamber that is used to perform antigen retrieval at various discreet temperatures. After completing the protocol, allow for the DIVA solution to cool down to RT before proceeding to the next step. Placing the DIVA solution in an ice bath can greatly expedite the cooling time. After solution reaches RT, wash slides twice in TBS for 5 min each. Carefully transfer the slides from the slide staining rack into a slide staining plate with cover lid (CL-SSP-20, AMScope). Ensure that the bottom of the slide staining plate is covered with a modest amount of water to keep the inside of the plate moist and prevent slides from drying out at any point during the staining procedure. To block endogenous peroxidase activity, pipette 200 μl of 3 % hydrogen peroxide homogenously across the entire slide and incubate slides with lid on the staining plate for 10 min. Higher concentration of hydrogen peroxide than recommended can greatly compromise the quality of the tissue. Remove excess hydrogen peroxide by washing, or briefly dipping the slides in TBS. Following the quick wash, treat slides with 200 μl of Levamisole for 20 min in the same fashion as described above. Levamisole is a competitive inhibitor of alkaline phosphatase (AP), which reduces background generated by endogenous AP activity. Following incubation, rinse slides twice for 5 min each in TBS. To reduce non-specific binding of antibody to antigens, prepare a working 10 % donkey-based blocking solution (10 % Donkey Serum+0.3 % 100X-Triton in TBS). Pipette 200 μl of the blocking solution over the tissue and carefully place pre-cut parafilm over the tissue and incubate for 1 h at RT. Application of parafilm helps the solution evenly disperse throughout the entire surface of the tissue, thereby reducing the amount of solution and antibody needed per slide. Calbindin Primary Antibody Incubation: Prepare the CB primary antibody solution by diluting the desired antibody concentration (1:200, Swant 300,Switzerland) in a donkey-based blocking solution (Table 1). Remove excess blocking and add 200 μl of antibody solution and cover slides with parafilm (S37440, Bemis Company Inc, USA). Incubate slides in dark humid box with lid overnight at 4 °C or RT.

### 3.2 Day 2: first primary antibody development, and second primary antibody incubation

Secondary Antibody Incubation: Following overnight incubation with primary antibody, gently remove parafilm from slides, and rinse in TBS, twice for 5 min each. Prepare biotinylated anti-Mouse IgG secondary antibody solution (1:200, 715-065-150, Jackson Laboratories,USA) in 10 % donkey blocking buffer (Table 1). The biotinylation of this conjugated secondary antibody allows for large complexes of biotin molecules to accumulate. We recommend using a 1:200 secondary antibody concentration, but this concentration may vary based on target protein. Pipette 200 μl of desired secondary antibody solution over the tissue and cover with parafilm for 2 h at RT.

ABC Incubation: Prepare the ABC solution using the manufacturers' protocol (9 μl reagent A + 9 μl reagent B, in 1 ml of diluent) at least 30 min prior to use. The ABC solution serves to further amplify the biotin signal on the secondary antibody. Pipette 200 μl of ABC solution over the tissue and cover with parafilm for 2 h at RT.

Tissue Development with DAB: Rinse slides in TBS twice for 5 min each and prepare DAB solution according to manufacturers' protocol. Hydrogen Peroxide (H_2_O_2_) oxidizes DAB in a reaction catalyzed by HRP. At the sites of HRP, the oxidized DAB forms a brown-colored product. It is important to only develop tissue with DAB until brown reaction product is achieved, usually within 1–3 min, as developing too long can result in dark tissue appearance that overshadows positive signal. To stop DAB developing, quickly rinse slides twice in TBS for 5 min each. After rinsing off excess DAB, pipette 200 μl of 10 % donkey-based blocking buffer over the slides and cover with parafilm for 2 h at RT.

Calretinin Primary Antibody Incubation: Prepare CR primary antibody solution (1:200. Swant 7697) by diluting the primary antibody (1:200, Swant 7697,Swtizerland) in 10 % donkey-based blocking buffer (Table 1). Pipette 200 μl of primary antibody solution and carefully cover the slides with parafilm. Incubate slides overnight at RT.

**Table 1:** Antibody Table. Detailed table summarizing the information related to the antibodies used in this protocol and their respective concentrations used.

Antibody (AB) Name:	Primary or Secondary AB?	Conjugated with Enzyme?	Species AB was Raised in:	AB Concentration Used:	AB Catalog Number:	AB RRID:
Parvalbumin (monoclonal mouse anti-Parvalbumin)	Primary	N/A	Mouse	1:250	Swant 235	AB_10000343
Calbindin (Monoclonal mouse anti-CB-D28K)	Primary	Not Applicable	Mouse	1:250	Swant 300	AB_10000347
Calretenin (polyclonal rabbit anti-Calretenin)	Primary	Not Applicable	Rabbit	1:250	Swant 7697	AB_2619710
Biotin-SP (long spacer) AffiniPure Donkey Anti-Mouse IgG (H + L)	Secondary	Biotin	Donkey	1:250	715-065-150	AB_2307438
Alkaline Phosphatase AffiniPure Donkey Anti-Rabbit IgG (H + L)	Secondary	Alkaline Phosphatase	Donkey	1:250	711-055-152	AB_2340591

### 3.3 Day 3: second primary antibody development, and third primary antibody incubation

Secondary Antibody Incubation: Following overnight incubation with CR primary antibody, carefully remove parafilm and rinse slides in TBS twice for 5 min each. Prepare AP conjugated secondary antibody solution (1:250) in 10 % donkey blocking buffer (Table 1). Pipette 200 μl of secondary antibody solution over the tissue and cover with parafilm for 2 h at RT. Rinse slides twice in TBS for 5 min each.

AP Vector Blue Tissue Development: Prepare AP Vector Blue solution according to manufacturers' protocol. Specifically, 2 drops (80 μl) of Vector Blue Reagent 1, Vector Blue Reagent 2 and Vector Blue Reagent 3 are added to 5 ml of 200 mM Tris-HCL pH8.2–8.5 buffer to create the buffered solution. The secondary antibody is bound to the AP detection enzyme, reacts with activating molecules found in Vector Blue Reagents 1–3 to form an insoluble, dark blue colored reaction product. While the activator molecule is not specified by the manufacturer, other AP based kits use known substrates and activators. For example, the Blue-Color AP Staining kit (AP100B-1, Systems Bio, USA) is a histochemical assay where AP hydrolyzes the phosphate group on the substrate, 5-Bromo-4-chloro-3 indolyl phosphate (BCIP) to form a blue colored intermediate. Nitro Blue Tetrazolium (NBT) then oxidizes the intermediate to form an insoluble, dark blue dimer, similar to the product we achieve in our protocol. Carefully develop the tissue with AP blue solution until a blue reaction product is achieved, usually within 5–10 min. Depending on the antibody concentration used, the duration of this step may vary. To stop the reaction, quickly rinse slides twice in TBS for 5 min each. After rinsing off excess AP blue solution, pipette 200 μl of 10 % donkey-based blocking buffer over the slides and cover with parafilm for 2 h at RT.

Avidin Biotin Blocking: Following this incubation, it is imperative to perform an Avidin/Biotin blocking step to block all endogenous biotin, as well as biotin receptors and avidin binding sites that were left unbound from the previous HRP reaction in day 2 of the protocol. First incubate tissue with reagent A (Avidin solution) for 15 min, followed by a quick TBS wash and 15-min incubation with reagent B (Biotin solution).

Parvalbumin Primary Antibody Incubation: After performing another quick TBS wash, prepare PV primary antibody solution (1:250, Swant 235) by diluting in 10 % donkey-based blocking buffer (Table 1). Pipette 200 μl of primary antibody solution and carefully cover the slides with parafilm. Incubate slides overnight at RT.

### 3.4 Day 4: third primary antibody development, and final steps

Secondary Antibody Incubation: Following overnight incubation with PV antibody, carefully remove parafilm and rinse slides in TBS twice for 5 min each. Prepare biotinylated anti-mouse IgG secondary antibody solution (1:200, 715-065-150, Jackson Laboratories,USA) in 10 % donkey blocking buffer (Table 1). Pipette 200 μl of secondary antibody solution over the tissue and cover with parafilm for 2 h at RT. Remove parafilm and rinse the slides in TBS twice for 5 min each.

ABC Incubation: Prepare ABC solution using the manufactures protocol (9 μl reagent A + 9 μl reagent B in 1 ml of diluent) at least 30 min prior to use. Pipette 200 μl of ABC solution over the tissue and cover with parafilm for 2 h at RT. Rinse slides in TBS twice for 5 min each and prepare the Vector-VIP HRP solution according to manufacturers' protocol. This solution produces an intense violet (purple) reaction.

Vector VIP Tissue Development: As with the other enzymatic reactions, it is important to only develop tissue with Vector VIP solution until violet reaction product is achieved, usually within 5 min, as developing too long can result in dark tissue appearance that overshadows positive signal. To stop Vector VIP developing, quickly rinse slides twice in TBS for 5 min each.

Tissue Dehydration and Cover-slipping: To finalize, dehydrate tissue in successive baths of 50 % (30 s), 70 % (4 min), 95 % (5 min) and 100 % (5 min) ethanol, and clear tissue with two 5-min incubations in Xylene. Lastly, coverslip slides with Permount or similar solution (SP15-100, Fisher Scientific, USA), ensuring that there are no air bubbles trapped underneath the coverslip. Allow slides to fully dry overnight prior to analysis.

### 3.5 Reagents

• TBS – (6.05 g Tris and 8.76 g NaCl in H_2_O, adjust to 1L, pH 7.5)
• PBS – (0.245g KH_2_PO_4_,1.44g Na_2_HPO_4_,0.2g KCL,8g NaCL, pH 7.4)
• Decloaking Chamber (DV2004, LX, MX, BioCARE Medical, USA)
• 10 % DIVA Decloaking Solution: (DV2005 L2J, BioCARE Medical, USA) 50 ml 100 % DIVA decloaking solution + 450 ml deionized water
• Levamisol (SP-5000-18, Vector Laboratories)
• 3 % Peroxidase Solution (6PMX3, Spectrum Chemical, USA) (100 μl of 30 % H_2_O into 10 ml DI H_2_O.)
• Donkey Blocking Solution (D9663, Sigma Aldrich, USA) (10 % Donkey Serum +0.3 % Triton X-100 in TBS)
• Permount Mounting Solution (SP15-100, Fisher Scientific, USA)
• VECTASTAIN Elite ABC Kit, Peroxidase, Standard (PK-6100, Vector Laboratories)
• Avidin-Biotin Blocking Kit (SP-2001,(Vector Laboratories)
• DiaminoBenzoicAcid (DAB) substrate (Vector Laboratories)
• Vector Blue Substrate Kit, Alkaline Phosphatase (SK-5300, Vector Laboratories)
• Vector-VIP Substrate Kit, Peroxidase (SK-4600, Vector Laboratories)
• Primary Antibodies: Monoclonal mouse anti-CB-D28K (Swant 300,Switzerland), polyclonal rabbit anti-Calretenin (Swant 7697,Swtizerland), and monoclonal mouse anti-Parvalbumin (Swant 235, Switzerland)
• Secondary Antibodies: Donkey Anti-Mouse conjugated with Biotin (715-065-150, Jackson Laboratories,USA) and Donkey anti-rabbit antibody conjugated with alkaline Phosphatase (711-055-152, Jackson Laboratories, USA).
• Cover Glass slides (22-038-105, Fisher Scientific,USA)
• Donkey Serum (D9663-10 ML, Sigma Aldrich)
• 200 Proof Pure Ethanol (DSP-MD-43, Avantor, USA)
• Xyelene (A5597, Sigma Aldrich, USA)
• Chloroform (c606SK-4, Fisher Scientific, USA)
• Triton X-100 (9036-19-5, Sigma Aldrich,USA)
• Parafilm M Wrapping Film (S37440, Bemis Company Inc, USA)

## 4 Results

**Fig. 1:**
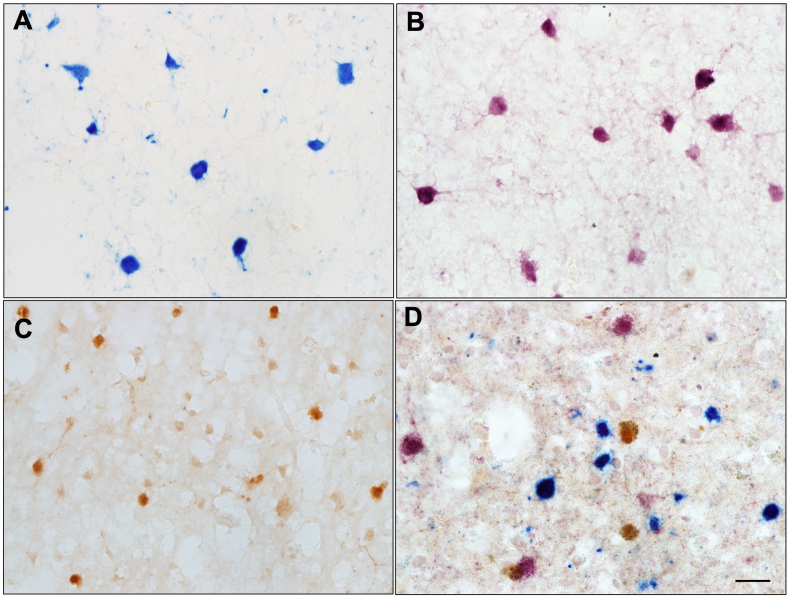
Human somatosensory cortex (BA3) immunostained with antibodies against parvalbumin (PV, pink), calretinin (CR, blue), and calbindin (CB, brown). Single IHC of CR + interneurons (A), PV + interneurons (B) and CB + interneurons (C), and triple IHC of PV+, CR+ and CB + interneurons (D). Almost all GABAergic interneurons express one of three Calcium Binding Proteins (CB,CR,PV) in a non-overlapping fashion. Scale bar = 20 μm.

**Fig. 2:**
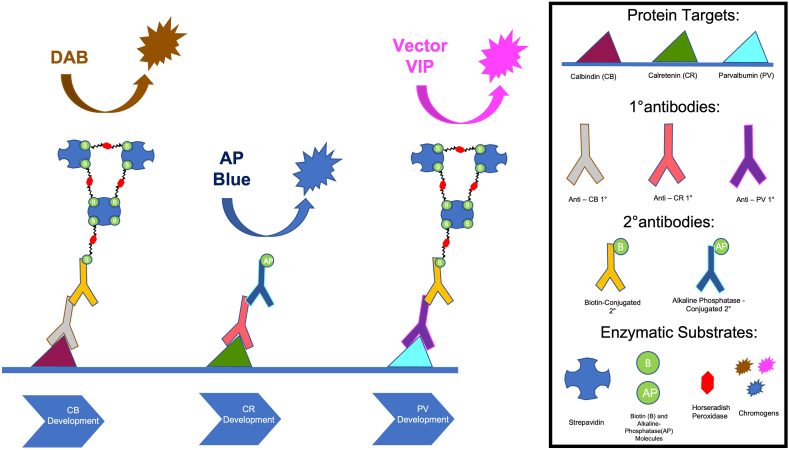
Sequential IHC of three antigens: calretinin (CR), calbindin (CB), and parvalbumin (PV), using our triple enzymatic IHC protocol. Each antigen was bound by a primary antibody and a secondary antibody that was conjugated with a biotin (B) or alkaline phosphatase (AP) enzyme. Biotin based detection system (Horseradish Peroxidase and Streptavidin) was used to detect CB and PV on days 2 and 4 of the protocol, and an AP based detection system was used to detect the CR on day 3 of the protocol. CB was developed with DAB to produce a brown color, CR was developed with Vector blue to produce a blue color, and PV was developed with Vector VIP to produce a pink color.

We employed triple enzymatic immunostaining to detect interneuron populations in postmortem human cerebral cortex. To optimize the triple stain method for the PV/CB/CR interneuron populations, we first performed single IHC to detect each of the proteins and decided what was the best protocol to detect cells expressing each protein (Fig. 1A–C). Then we merged protocols, tested a variety of combinations of colors for each protein, and chose to proceed with the one that rendered the best result. The best result was given by the protocol that achieved the most robust cellular label and clear contrast for each individual cell population. Negative control experiments were run by eliminating the primary antibody from the protocol. Obtaining clear visual contrast between signals is imperative for accurate quantification of cells in double or triple enzymatic IHC (Fig. 2). We determined that the combination of DAB-brown for CB, AP-blue for CR, and Vector VIP-pink for PV was the combination that allowed to quantify the highest number of labeled cells per marker (Fig. 1D). This triple enzymatic IHC method was first used in one of our prior studies where we assessed for alterations in cortical GABAergic interneuron populations in the prefrontal cortex of brains from subjects with autism [10]. The quantification results described below are largely based on those that were previously reported in this study. We quantified specific interneurons and calculated the ratio of interneuron subtypes in several areas of the prefrontal cortex (BA46, BA47, and BA9) in autism and control brains, and determined that there was a significant decrease of PV + interneurons, but not other interneuron types (CB+,PR+), in autism when compared to control cortex in the three areas of interest [10]. Most positive cells expressed only one protein. The number of clearly double-labeled cells was minimal across subjects, with <1 % of interneurons expressing two proteins. Occasionally, we observed a cell that expressed the three proteins. There was a small number of CB + cells (<0.01 %) cells that based on morphology were identified as pyramidal neurons in BA's 9, 46 and 47 of the prefrontal cortex, as previously reported [10].

**Fig. 3:**
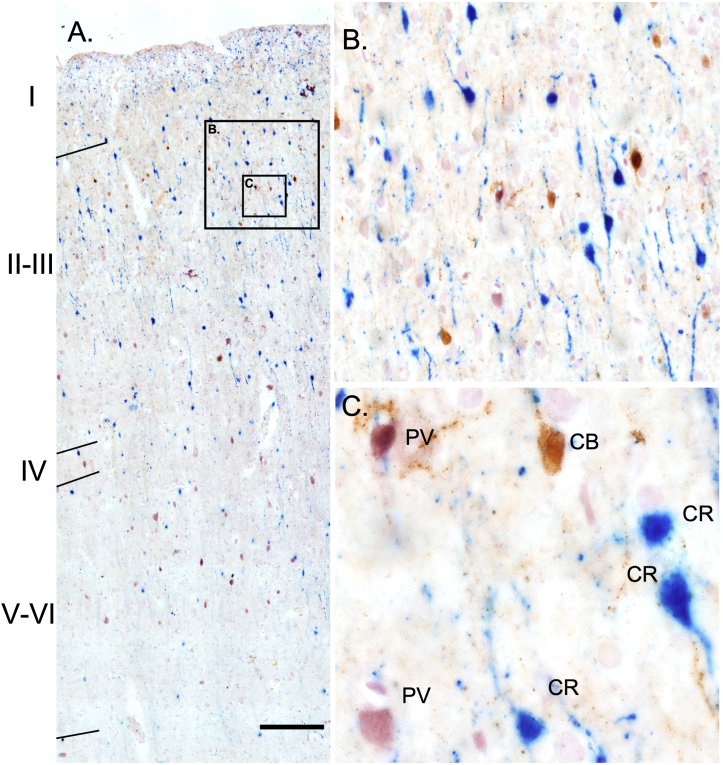
Anatomical Distribution of PV+, CB+ and CR + cells across layers I-VI in BA46 of the prefrontal cortex using triple enzymatic IHC. PV+ (Pink), CB+ (Brown) and CR+ (Blue) cell densities can be appreciated in the supragranular layers (I-III) and the infragranular layers (V-VI) (A). Layer 2 specific distribution (B) and morphological characteristics of PV+, CB+ and CR + cells are depicted(C). Scale Bar = 200 μm.

CB + interneuron number was higher in the supragranular layers (I-III) than in the infragranular layers (V/VI) across all three areas analyzed (Fig. 3A). CB + interneurons innervate the distal dendritic arbor of pyramidal neurons. Many of these cells had horizontally spreading processes, with multipolar and bipolar dendrites that arborized in layer I (Fig. 3C), all features consistent with Martinotti cells [[12], [13], [14]]. CR + interneurons were more abundant in the supragranular layers than in the infragranular layers across all three areas (Fig. 3A). Based on morphology and location, upper layer CR + interneurons appeared to be bipolar interneurons [[15], [16], [17]]. CR + bipolar interneurons have intracolumnar, vertically orientated dendrites across many layers (Fig. 3B). Interneurons immunoreactive for PV were either basket (Bsk) cells or chandelier (Ch) cells [12,18,19]. The laminar distribution of PV + cells spanned across layers II-VI, with more neurons in the supragranular layers (Fig. 3A). Ch cells have multipolar axonal processes that are local and exclusively target the axonal initial segment of pyramidal cells. Bsk cells are the most abundant PV + interneuron subtype and are characterized as having a multipolar dendritic arbor with an axon that target pyramidal soma and other PV+ and CR + interneurons [12,18,19].

## 5 Discussion

Multiple IHC is a powerful tool that makes possible the simultaneous detection of two or more antigens on the same tissue section. Multiple IHC can be carried out as a simultaneous or sequential multiday protocol. While simultaneous protocol is much more time efficient, the sequential multiday protocol provides better specificity for detecting the target antigens. The success of the protocol relies on multiple factors, most notably the choice of antibodies that result with the least amount of cross reactivity and the choice of chromogenic reagents that provide the highest contrast. Here we considered all these conditions to develop a successful triple enzymatic IHC protocol to detect 3 distinct protein markers in formalin fixed human tissue.

### 5.1 Monoclonal vs polyclonal antibody

There are two types of antibodies used in IHC, polyclonal and monoclonal. Polyclonal antibodies are a heterologous mixture of IgGs against multiple epitopesonthe antigen, while monoclonal antibodies are composed of a single IgG against a single epitope. Polyclonal antibodies are characterized by a greater antibody affinity and are more tolerant to small changes of the protein structure caused by denaturation or dimerization. However, high affinity decreases specificity since it can recognize a similar amino acid sequence on a non-target antigen, rendering cross reactivity and background. Also, batch-to-batch variability exist since they are produced in different animals. Despite this, affinity purified antibodies can minimize this effect. Polyclonal antibodies are commonly used when attempting to detect proteins whose concentration is low in the tissue. Another advantage of polyclonal antibodies is that are cheaper and faster to produce. Conversely, monoclonal antibodies have higher specificity since they recognize a single epitope on an antigen. This increased specificity translates into a lower cross reactivity and background. Monoclonal antibodies are produced *ex-vivo* in hybridomas and are batch-to-batch reproducible, allowing large quantities of identical antibody. However, they are more expensive and take a longer time to produce. Monoclonal antibodies can be used for many purposes, being ideal for identification of specific sequences of a protein, or for assays requiring quantification of protein levels. In our study, the use of polyclonal antibodies was more suitable due to the ability of the antibody to recognize multiple epitopes on the antigen.

### 5.2 Antibody species

The number of antigens to be detected in a single tissue slice is largely limited by the available number of primary antibodies generated in different animal species. It is highly recommended to use primary antibodies raised in different species. Using two primary antibodies generated in the same species can increase the possibility of a secondary antibody binding to more than one of these primary antibodies. This results in nonspecific signal and compromises the ability to accurately identify distinct antigens. While most studies use antibodies raised in different species, it is possible to use antibodies generated in the same species. Published work have successfully use combination of two mouse-generated antibodies in both enzymatic and fluorescence based techniques [10,20,21]. The first study used heat-induced epitope retrieval (HIER) to block the first antibody before the application of the second antibody from the same host species. However, only tonsil and kidney formalin fixed tissue was used in this study, so protein abundance may differ from that seen in brain tissue. Another study used multiple immunolabeling with multiple antibodies from the same host species to achieve clear signal in human breast, skin, small intestine and tonsil tissues from human patients [21]. They did so by using stripping buffers that eluted the primary/secondary antibody complex. Like the last study, no brain tissue was stained and there is also the added limitation of susceptibility to photobleaching and long-term preservation of tissue, that we discuss below. The method we describe here in detail last used in our previous published study [10] was possible due in part to the use of a commercially available avidin-biotin blocking reagent (SP-2001, Vector Laboratories). This method is generally used to block endogenous biotin in the tissue, but it also can be used to block exogenous biotin product that was applied to the tissue during an IHC procedure. First streptavidin is added to bind all endogenous biotin molecules. Free biotin (Biotin solution) is then added to the tissue to block excess biotin-binding sites on the avidin. This ensures that all endogenous molecules are bound by avidin and all biotin-binding sites on the avidin are bound by biotin. When developing our protocol, we found that blocking biotin molecules from the ABC complex from day 2 of our procedure greatly reduced the amount of potential cross reactivity that may occur by using a second secondary antibody from the same host species, that also uses a HRP enzyme catalyzed reaction, in day 4 of our protocol.

### 5.3 Direct vs indirect IHC

IHC can be performed using a direct or indirect method. The direct method allows for visualization of a protein using a primary antibody conjugated to a color producing molecule. Direct detection is more cost and time efficient as it eliminates the need for an additional amplification step with a secondary antibody. There is also less chance of non-specific signal amplification coming from a secondary antibody. Direct detection is more suitable for use in tissue where the target antigen is highly abundant, as a weak signal will result when the target antigen is not abundant. The indirect method utilizes a conjugated secondary labeled antibody to detect the target protein. This method is more suitable for targeting an antigen that is not abundant, however the indirect method is more costly and less time efficient as it requires extra steps. Also, the indirect method may generate more non-specific signal, due to secondary antibody binding to non-target primary antibodies and proteins. While both methods have benefits and drawbacks, the choice of using direct or indirect detection is often determined by the overall abundance of the target antigen within the tissue of interest. Our protocol benefits from the use of an indirect method. By using the extra amplification step to increase our signal, the contrast between our multiple markers improves allowing for more feasible cell quantification.

### 5.4 Fluorescent vs enzymatic IHC

Fluorescent IHC provides visualization of an antigen using a fluorochrome conjugated antibody. Given the large number of commercially available fluorochromes, simultaneous detection of multiple antigens can be achieved. Fluorophores emit lights of specific wave lengths allowing for independent and overlapping visualization of antigens. While immunofluorescence has multiple advantages, it also has pitfalls. Photobleaching is the most notable limitation that results from the fluorescent signal becoming weaker on the tissue after every use under the microscope. This occurs when illumination from the microscope's laser causes the fluorophore to permanently change its molecular structure, inhibiting it from emitting light at its highest excitation state. While a signal amplification step may help to ameliorate this problem acutely, long term use over time will decrease the fluorescent signal. This could potentially affect studies that require extensive quantification, such as ours. In instances where fluorescent signal is decreased due to photobleaching of the tissue, quantification may be compromised and the results may not be representative of true biological changes occurring, rather they may be the result of artificially induced changes that occur due to photobleaching. Another common challenge faced when staining non-perfused human tissue is high levels of autofluorescence or non-specific signal. The amount of autofluorescence increases with higher levels of postmortem intervals, the time from death to fixative immersion. Erythrocytes are auto fluorescent because hemoglobin absorbs wavelengths under 600 nm. Another source of autofluorescence is lipofuscin. Lipofuscin is a lipid residue of lysosomal digestion that accumulates forming yellow pigmented granules. Lipofuscin granules accumulate in the cytoplasm of cells during aging. Lipofuscin granules autofluorescence peaks at 435 nm. It is more abundant in aged individual and is sometimes called the “aging pigment” due to its linear accumulation with increasing age [[22], [23], [24]]. This endogenous autofluorescence makes it difficult to accurately distinguish the experimental fluorescent signal. Techniques such as treating non-perfused tissue with Sudan Black B dye or TrueBlack reduce the non-specific signal, but it does not completely mask it [25]. While this study has successfully used an IF technique to stain formalin fixed human tissue, the limitation of long-term preservation of fluorescent signal remains.

In a different mechanism, enzymatic IHC detects an antigen using an enzyme that transforms a soluble substrate into a colored precipitate product at the antigen site. Chromogens are significantly more resistant to photobleaching and can be preserved for indefinite periods of time relative to an immunofluorescent IHC. This makes enzymatic IHC ideal for detecting signal without photobleaching. Long term preservation of stained tissue allows the researcher to use it for multiple quantification purposes without having to re-stain again due to photobleaching, making it an overall more practical and cost-effective technique ,relative to IF, for the purposes of our study. While advantageous in many aspects, enzymatic IHC has some pitfalls, most notably difficulty in assessing for co-localization of two or more markers in the same cell structure. Chromogenic reaction products overlap when they co-localize in the same area and the microscopes cannot readily separate colors as in the case for fluorochromes. This makes fluorescent IHC better suited for colocalization of two target antigens within the same cellular structure. Multi-enzymatic staining, such as the protocol we present here, is more useful when detecting different cell populations. Another limitation is the number of fewer colors available relative to the fluorescent counterparts, limiting the number of chromogens that can be used. When establishing a protocol for triple enzymatic IHC it is essential to choose the sequence of antibody/chromogen application that will yield the optimal signal. Choosing chromogens that yield different color precipitates with very different contrast provides the clearest contrast differentiation. If there is sufficient contrast differentiation between colored precipitates produced, then colocalization may be observable between two different markers. If the color between two color precipitates is similar, colors may be confused by the observer. We recommend combining the chromogen in a specific sequential order to obtain the best contrast for each of the three signals. Applying DAB reaction product in the first staining sequence is imperative for obtaining successful multi-staining results. DAB's is the only known chromogen that induces a “shielding” effect which results in effective shielding of the immunoreagent used in the first staining procedure [26]. That is, the brown DAB precipitate that forms from the development reaction at the end of the second day of our protocol is able to shield other targets used in second and third IHC staining sequences. This shielding phenomenon can help to prevent any potential cross-reaction reaction with other reagents, such as vector VIP, which also use an HRP based reaction. Together with the Avidin, Biotin Block, the DAB shielding effect allows us to effectively use two HRP based reactions to produce clear and distinct signal for two different antigens on the same tissue. We determined that the combination of DAB-brown for CB, AP-blue for CR, and Vector VIP-pink for PV was the best sequential multi day staining combination that allowed us to obtain clearest contrast to quantify the highest number of labeled cells per marker with almost no cross reactivity.

## 6 Conclusion

Immunostaining is a very useful tool that help us to understand biological processes. By using a combination of (AP) and HRP based enzymatic reactions we were able to successfully detect three distinct antigens in non-perfused human autism brain tissue. When developing this protocol, we considered the type of antibody used, detection system choice, chromogen choice, and sequential order of protocol. The combination of brown (DAB), blue (AP Blue) and pink (Vector VIP) are the three-color choices that provided the best contrast to distinguish the three different cortical cell populations. When adapting this protocol for use in human or animal tissue, we suggest individually optimizing each of the variables. Triple enzymatic IHC is a valuable tool that can allow us to better understand the complexity of different cellular, molecular, and anatomical alterations occurring in human tissue.

## 7 Detailed protocol

Triple enzymatic staining allows for staining of three different antigens on human and/or animal tissue. The following 4-day protocol has been optimized for staining slide mounted tissue 14 μm in thickness.

### 7.1 Day 1: Pretreatment of slides and incubation with first primary antibody

1. Thaw out frozen slides in a dry bath (37 °C) for 10 min prior to starting the staining procedure.
2. Place slides in an immunostaining slide staining rack.
3. Immerse slides in a 1:1 solution of 100 % Ethanol and Chloroform at room temperature, RT (20–22 °C) for 10 min as a defatting process.
4. Rehydrate tissue by immersing slides in successive baths of 100 % ethanol (5 min), 90 % ethanol (5 min), 70 % ethanol (4 min), 50 % ethanol (3 min) and DiH_2_O (30 s).
5. Rinse slides twice in TBS for 5 min each.
6. Perform Antigen Retrieval
a. Immerse slides in a 10 % DIVA decloaking solution for 5 min.
b. Place slides in Decloaking Chamber and follow manufactures quick Guide to setup the Antigen Retrieval protocol for 6 min at 100 °C (V.3.7.2.2).
c. Start the program and wait for DIVA solution to cool to RT before proceeding to the next step *

*Placing DIVA solution with slides over an ice bath can greatly expedite the cooling time reaching RT.

7. Rinse slides in TBS solution twice for 5 min each.
8. Remove slides from the slide staining rack and place them back on a slide staining plate with cover lid. Ensure to fill the bottom of the staining plate with water to prevent slides from drying out at any point during the staining procedure.
9. Treat slides with 3 % peroxidase for 10 min. Carefully pipette 200 μL of 3 % peroxidase homogenously across the entire slide and incubate with lid on for 10 min
10. Remove excess peroxidase by briefly dipping the slides in TBS.
11. Treat slides with 200 μL of Levamisole for 20 min in the same fashion as described above in step 9.
12. Remove slides from staining plate and place back on rack. Rinse slides in TBS solution twice for 5 min each.
13. Prepare a working 10 % donkey-based blocking solution (10 % Donkey Serum+0.3 % 100X-Triton in TBS).
14. Pipette 200 μL of the blocking solution over the tissue and carefully place pre-cut parafilm over the tissue and incubate for 1 h at RT.
15. Prepare 1°antibody solution by diluting the desired antibody concentration in a donkey-based blocking solution.
16. Remove excess blocking buffer by briefly dipping the slides in TBS
17. Pipette 200 μL of 1° antibody solution and parafilm slides.
18. Incubate the slides overnight at 4 °C or RT.

### 7.2 Day 2: application of biotinylated secondary antibody, development with DAB and application of second primary antibody

1. Following overnight incubation with 1° antibody, gently remove parafilm, and rinse with TBS twice for 5 min each.
2. Prepare Biotinylated Anti-Mouse IgG secondary antibody-based solution with desired concentration in 10 % donkey blocking buffer.
3. Pipette 200 μL of desired secondary antibody solution over the tissue and cover with parafilm for 2 h at RT.
4. Rinse slides in TBS twice for 5 min each.
5. Prepare Avidin-Biotin Complex (ABC) solution using the manufactures protocol (9 μL+ 200 μL ) *
a. *Make sure that ABC solution is prepared at least 30 min prior to use
6. Pipette 200 μL of ABC solution over the tissue and cover with parafilm for 2 h at RT
7. Rinse slides in TBS twice for 5 min each.
8. Prepare DAB solution according to manufactures protocol. Develop tissue with DAB until brown reaction product is achieved. Depending on the antibody and ABC concentrations used, the duration of this step may vary.
9. To stop DAB developing, rinse slides in TBS for 5 min each
10. Pipette 200 μL of 10 % donkey-based blocking buffer over the tissue and cover with parafilm for 2 h at RT.
11. Prepare 1°antibody solution by diluting the desired antibody concentration in 10 % donkey-based blocking buffer.
12. Pipette 200 μL of 1° antibody solution and carefully cover the slides with parafilm
13. Incubate slides overnight at 4 °C or RT.

### 7.3 Day 3: application of alkaline phosphatase (AP) conjugated secondary antibody, development of reaction with Vector Blue and application of third primary antibody

1. Following overnight incubation with 1° antibody, carefully remove parafilm and rinse slides in TBS twice for 5 min each.
2. Prepare Alkaline Phosphatase conjugated secondary antibody-based solution with desired concentration in 10 % donkey blocking buffer.
3. Pipette 200 μL of desired secondary antibody solution over the tissue and cover with parafilm for 2 h at RT.
4. Rinse slides twice in TBS for 5 min each.
5. Prepare AP Vector Blue solution according to manufactures protocol. Develop the tissue with AP blue solution until a blue reaction product is achieved. Depending on the antibody and ABC concentrations used, the duration of this step may vary.
6. Rinse the slides in TBS three times for 5 min each to stop the AP Blue developing step.
7. Pipette 200 μL of donkey-based blocking solution over the tissue and cover with parafilm.
8. To block endogenous Avidin and Biotin use the Avidin-Biotin blocking kit following the manufactures protocol.
9. Quick wash slides in TBS and prepare 1°antibody solution by diluting the desired antibody concentration in 10 % donkey blocking buffer.
10. Pipette 200 μL of 1° antibody solution and carefully cover the slides with parafilm
11. Incubate the slides overnight at 4 °C or RT.

### 7.4 Day 4: application of biotinylated donkey anti-mouse secondary antibody, development of vector-VIP and dehydration of slides

1. Carefully remove parafilm and rinse the slides in TBS twice for 5 min each.
2. Prepare Biotinylated Anti-Mouse IgG secondary antibody-based solution in 10 % donkey-blocking buffer to desired concentration.
3. Pipette 200 μL of 2° antibody solution over the tissue, carefully cover with parafilm and incubate at RT for 2 h.
4. Carefully remove parafilm and rinse the slides in TBS twice for 5 min each.
5. Prepare Avidin-Biotin Complex (ABC) solution using the manufactures protocol*
a. *Prepare ABC solution at least 30 min prior to incubation
6. Pipette 200ul of ABC solution over the tissue and cover with parafilm for 2 h at RT.
7. Prepare Vector VIP developing solution according to manufactures protocol. Develop the tissue with Vector VIP until an intense violet reaction product is achieved. Depending on the antibody and ABC concentrations used, the duration of this step may vary.
8. Rinse the slides in TBS three times for 5 min each to stop the Vector VIP developing step.
9. Dehydrate the tissue by immersing slides in successive baths of 50 % ethanol (30s), 70 % ethanol (4 min), 95 % (5 min), 100 % (5 min) followed by a 10-min incubation of Xylene (2 times, 5 min each).
10. Coverslip slides with permount solution and allow to fully dry overnight prior to using.

## Author contribution statement

Pablo Juarez: Performed experiments; Analyzed and interpreted data: Wrote the paper.

Verónica Martínez-Cerdeño: Conceived and designed the experiments; Analyzed and interpreted the data; Contributed reagents, and wrote the paper.

## Data availability statement

Data is available in the supplementary material and can be made available upon reasonable request.

## Declaration of competing interest

The authors declare that they have no known competing financial interests or personal relationships that could have appeared to influence the work reported in this paper.
