# Supplementary material for: Triple enzymatic immunochemistry for interneuron populations in postmortem human cerebral cortex
Source: Heliyon. 2023 Oct 11;9(10):e20626. doi: 10.1016/j.heliyon.2023.e20626 (PMC10589777; doi:10.1016/j.heliyon.2023.e20626)
Supplement: Multimedia component 1 [file mmc1.docx]

**Bench Protocol**

Triple enzymatic staining allows for staining of three different antigens on human and/or animal tissue. The following 4-day protocol has been optimized for staining slide mounted tissue 14 μm in thickness.

Day 1: Pretreatment of Slides and Incubation with First Primary Antibody

1. Thaw out frozen slides in a dry bath (37°C) for 10 minutes prior to starting the staining procedure.
2. Place slides in an immunostaining slide staining rack
3. Immerse slides in a 1:1 solution of 100% Ethanol and Chloroform at room temperature, RT,(37°C) for 10 minutes as a defatting process.
4. Rehydrate tissue by immersing slides in successive baths of 100% ethanol (5 minutes), 90% ethanol (5 minutes), 70% ethanol (4 minutes), 50% ethanol (3 minutes) and DiH_2_O (30 seconds).
5. Rinse slides twice in TBS for 5 minutes each
6. Perform Antigen Retrieval
   1. Immerse slides in a 10% DIVA decloaking solution for 5 minutes.
   2. Place slides in Decloaking Chamber and follow manufactures quick Guide to setup the Antigen Retrieval protocol for 6 minutes at 100°C (V.3.7.2.2).
   3. Start the program and wait for DIVA solution to cool to RT before proceeding to the next step *

*Placing DIVA solution with slides over an ice bath can greatly expedite the cooling time.

1. Rinse slides in TBS solution twice for 5 minutes each.
2. Remove slides from the slide staining rack and place them back on a slide staining plate with cover lid. Ensure to fill the bottom of the staining plate with water to prevent slides from drying out at any point during the staining procedure.
3. Treat slides with 3% peroxidase for 10 minutes. Carefully pipette 200µL of 3% peroxidase homogenously across the entire slide and incubate with lid on for 10 minutes
4. Remove excess peroxidase by briefly dipping the slides in TBS.
5. Treat slides with 200µL of Levamisole for 20 minutes in the same fashion as described above in step 9
6. Remove slides from staining plate and place back on rack. Rinse slides in TBS solution twice for 5 minutes each
7. Prepare a working 10% donkey-based blocking solution (10% Donkey Serum+0.3% 100X-Triton in TBS)
8. Pipette 200µL of the blocking solution over the tissue and carefully place pre-cut parafilm over the tissue and incubate for 1 hour at RT.
9. Prepare 1°antibody solution by diluting the desired antibody concentration in a donkey-based blocking solution.
10. Remove excess blocking buffer by briefly dipping the slides in TBS
11. Pipette 200µL of 1° antibody solution and parafilm slides
12. Incubate the slides overnight at 4°C.

Day 2: Application of Biotinylated Secondary Antibody, development with DAB and Application of Third Primary Antibody.

1. Following overnight incubation with primary antibody, gently remove parafilm, and rinse with TBS twice for 5 minutes each.
2. Prepare Biotinylated Anti-Mouse IgG secondary antibody-based solution with desired concentration in 10% donkey blocking buffer.
3. Pipette 200µL of desired secondary antibody solution over the tissue and cover with parafilm for two hours at RT.
4. Prepare Avidin-Biotin Complex (ABC) solution using the manufactures protocol (9µL+ 200µL *
   1. *Make sure that ABC solution is prepared at least 30 minutes prior to use
5. Pipette 200µL of ABC solution over the tissue and cover with parafilm for two hours at (RT)
6. Rinse slides in TBS twice for 5 minutes each.
7. Prepare DAB solution according to manufactures protocol. Develop tissue with DAB until brown reaction product is achieved. Depending on the antibody and ABC concentrations used, the duration of this step may vary.
8. To stop DAB developing, rinse slides in TBS for 5 minutes each
9. Pipette 200µL of 10% donkey-based blocking buffer over the tissue and cover with parafilm for two hours at RT.
10. Prepare 1°antibody solution by diluting the desired antibody concentration in 10% donkey-based blocking buffer.
11. Pipette 200µL of 1° antibody solution and carefully cover the slides with parafilm
12. Incubate slides overnight at 4°C.

Day 3 : Application of Alkaline Phosphatase (AP) conjugated Secondary Antibody, Development of Reaction with Vector Blue and Application of Third Primary Antibody

1. Following overnight incubation with primary antibody, carefully remove parafilm and rinse slides in TBS twice for 5 minutes each.
2. Prepare Alkaline Phosphatase conjugated secondary antibody-based solution with desired concentration in 10% donkey blocking buffer.
3. Pipette 200µL of desired secondary antibody solution over the tissue and cover with parafilm for two hours at RT.
4. Rinse slides twice in TBS for 5 minutes each.
5. Prepare AP Vector Blue solution according to manufactures protocol. Develop the tissue with AP blue solution until a blue reaction product is achieved. Depending on the antibody and ABC concentrations used, the duration of this step may vary.
6. Rinse the slides in TBS three times for 5 minutes each to stop the AP Blue developing step
7. Pipette 200 µL of donkey-based blocking solution over the tissue and cover with parafilm.
8. To block endogenous Avidin and Biotin use the Avidin-Biotin blocking kit following the manufactures protocol.
9. Quick wash slides in TBS and prepare 1°antibody solution by diluting the desired antibody concentration in 10% donkey blocking buffer.
10. Pipette 200µL of 1° antibody solution and carefully cover the slides with parafilm
11. Incubate the slides overnight at 4°C.

Day 4: Application of Biotinylated donkey anti-mouse secondary antibody, development of vector-VIP and dehydration of slides.

1. Carefully remove parafilm and rinse the slides in TBS twice for 5 minutes each.
2. Prepare Biotinylated Anti-Mouse IgG secondary antibody-based solution in 10% donkey- blocking buffer to desired concentration.
3. Pipette 200µL of secondary antibody solution over the tissue, carefully cover with parafilm and incubate at RT for two hours.
4. Carefully remove parafilm and rinse the slides in TBS twice for 5 minutes each.
5. Prepare Avidin-Biotin Complex (ABC) solution using the manufactures protocol*
   1. *Prepare ABC solution at least 30 minutes prior to incubation
6. Pipette 200ul of ABC solution over the tissue and cover with parafilm for two hours at (RT)
7. Prepare Vector VIP developing solution according to manufactures protocol. Develop the tissue with Vector VIP until an intense violet reaction product is achieved. Depending on the antibody and ABC concentrations used, the duration of this step may vary.
8. Rinse the slides in TBS three times for 5 minutes each to stop the Vector VIP developing step
9. Dehydrate the tissue by immersing slides in successive baths of 50% ethanol (30s), 70% ethanol (4 minutes), 95% (5 minutes), 100% (5 minutes) followed by a 10-minute incubation of Xylene (2 times, 5 minutes each).
10. Coverslip slides with permount solution and allow to fully dry overnight prior to using.
